# A Treatise on Insanity, and Other Disorders of the Mind

**Published:** 1835-04-01

**Authors:** 


### A TREATISE ON INSANITY, AND OTHER DISORDERS AFFECTING THE MIND. *By J. C. Prichard*, M.D. &c. Octavo, pp. 483.

THOUGH the attention of Dr. Prichard appears to have been directed to the subject of insanity for twenty years past, yet it was the construction of the article "INSANITY," in the Cyclopædia of Practical Medicine, that gave origin to the present work, which is an extension and completion of the said article. We have every reason to believe that the CYCLOPÆDIA may prove a kind of ALMA MATER, whence will issue many a monograph that would not otherwise have seen the light. It will thus prove useful in a twofold manner to the public—it will stimulate individual writers to greater exertion, and diffuse knowledge through a great mass of readers.

The work is dedicated to M. Esquirol, a physician who has dedicated great attention and many years to the investigation of this class of human afflictions. In his preface, Dr. P. assigns as one reason for the publication, a conviction that some notions generally prevalent, and sanctioned by the highest medical and legal authorities in this country, in respect to insanity, are not only erroneous, but the "sources of great practical evils." This is certainly a good reason for coming before the public; but there are very many reasons why a man of Dr. Prichard's talents and experience should devote his powers to the investigation of such a difficult subject as that under consideration. The work is divided into twelve chapters, each being subdivided into numerous sections. The ramifications of the discussion, indeed, are so multifarious, that the study of the subject appears to be a most formidable task, and many will be deterred from the undertaking. We shall only be able to notice a few of the early chapters in this article, reserving for next number a more copious analysis of this important work.

### CHAP. I.—DEFINITION—NOSOGRAPHY OF INSANITY.

Definitions of diseases have always proved great stumbling-blocks to nosologists. Insanity does not exhibit an exception. It has had various definitions, none of which will apply to all cases. The disturbances of the mental operations present very different phenomena in different forms of the disease, and they cannot be described in a collective statement, without first considering the principal varieties. These our author enumerates—demolishing, as he proceeds, the definitions of Locke, Cullen, Sauvages, Linnæus, Pinel, and others. The four principal varieties, then, are thus arranged.

"1. *Moral Insanity*, or madness consisting in a morbid perversion of the natural feelings, affections, inclinations, temper, habits, moral dispositions, and natural impulses, without any remarkable disorder or defect of the intellect or knowing and reasoning faculties, and particularly without any insane illusion or hallucination.

The three following modifications of the disease may be termed *Intellectual Insanity*, in contradistinction to the preceding form. They are severally :—

2. *Monomania*, or partial insanity, in which the understanding is partially disordered or under the influence of some particular illusion, referring to one subject, and involving one train of ideas, while the intellectual powers appear, when exercised on other subjects, to be in a great measure unimpaired.

3. *Mania*, or raving madness, in which the understanding is generally deranged; the reasoning faculty, if not lost, is confused and disturbed in its exercise; the mind is in a state of morbid excitement, and the individual talks absurdly on every subject to which his thoughts are momentarily directed.

4. *Incoherence*, or dementia. By some persons it may be thought scarcely correct to term this a form of insanity, as it has been generally considered as a result and sequel of that disease. In some instances, however, mental derangement has nearly this character from the commencement, or at least assumes it at a very early period. I am therefore justified in stating it, after Pinel, to be a fourth and distinct form of madness.

It is thus characterized by that justly celebrated writer:—'Rapid succession or uninterrupted alternation of insulated ideas, and evanescent and unconnected emotions; continually repeated acts of extravagance; complete forgetfulness of every previous state; diminished sensibility to external impressions; abolition of the faculty of judgment; perpetual activity.'" 7.

Few of our readers will fail to perceive that the first form—*moral insanity*—is applicable to nine-tenths of society—nay, to ninety-nine in the hundred! Who is it that is not sometimes affected by "a *morbid* perversion of the natural feelings, affections, inclinations, temper, &c. without any remarkable disorder or defect of the intellect, &c.?" Not one! This moral madness is the universal disease, or rather state of man, and is that to which the Roman bard alludes—"insanire omnes, &c." Then, again, between the 3d and 4th species, at least according to Pinel's definition, it will often be hard to draw the line of distinction. In the one case, the individual "talks absurdly on every subject"—in the other, the ideas are in rapid succession, or uninterrupted alternation. The *latter* appears to be only a higher degree of the *former*. Be this as it may, the author has wisely determined to substitute for a definition "a short nosography of the disease," or summing up of the characteristics of the different forms.

"We may, then, describe insanity as a chronic disease, manifested by deviations from the healthy and natural state of the mind, such deviations consisting either in a *moral perversion*, or a disorder of the feelings, affections, and habits of the individual, or in *intellectual derangement*, which last is sometimes partial, namely, in *monomania*, affecting the understanding only in particular trains of thought; or general, and accompanied with excitement, namely, in *mania*, or *raving madness*; or, lastly, confounding or destroying the connexions or associations of ideas, and producing a state of *incoherence*." 7.

The next four chapters are, accordingly, dedicated to "an accurate and tolerably complete description of the phenomena of insanity," as arranged under the above heads. We cannot follow our author through these sections. It is evident that, in the first, or moral insanity—the "*folie raisonnante*," or *rational madness*, of Pinel, Dr. P. describes a disease much graver in grade than what we would conceive by the definition already quoted. "The individual himself has been discovered to have suffered, in a former period of life, an attack of madness, of a decided character." "He has become an altered man"—"he is continually engaging in new pursuits"—his affections become totally perverted—manifesting dislike or even enmity towards his best friends—in short the man is deranged, though, before a jury or an inquisition, his answers will often be so pertinent as to puzzle the arbiters of his fate—including even the Doctors! These are the kinds of cases that bring scandal on our cloth by eliciting such contradictory evidence from different medical witnesses. And no wonder—for the disease or disorder is nothing more than a higher grade of that under which we every one labour, from the king on the throne to the beggar in the streets! We are strongly inclined, therefore, to agree with M. Georget, in terming this species the *first stage* of insanity—"the incubation of madness"—even although it may last many years, or through life, with running into monomania or general insanity—incoherence. In not a few of these cases of "moral insanity," the tendency to gloom and melancholy is a prominent feature. The faculty of reason is not manifestly impaired, yet sadness clouds all the prospects of life—the individual, though surrounded by comforts, and at peace with himself and all the world, yet becoming sorrowful and desponding. All things, present and to come, are involved in gloom, and persons thus affected (if of good education) often express poignant grief at the inaptitude which they experience to go through the active duties of life. They often feel a horror of suicide, to which, nevertheless, they seem to be prompted by some diabolical instinct or internal feeling. They are, therefore, fearful of being left a moment alone. Such cases often terminate in recovery. People of an opposite character give themselves up to *tædium velut* and morose disgust, loathing their existence—and sometimes putting an end to it by their own hands. The almost infinite variety of shades which this species of insanity exhibits is well and ably delineated by our author, and we must refer to the work itself all our readers who desire to have ample information on the subject.

The section (II.) on MONOMANIA, is ably handled by our author. All medical men are aware that patients, labouring under this form of insanity, evince some peculiar illusion or erroneous conviction as to a particular subject, and are incapable of reasoning correctly on any topic connected with that particular subject, though on all others they are rational enough. It was termed melancholia by Hippocrates, though it is by no means consistent with the fact, that this species of derangement is essentially of a gloomy character, or connected with sadness and despondency. Some patients of this description are proud and elated, fancying themselves kings or emperors, and appearing to be really happy in their illusions. Melancholia originally meant what monomania now infers. Dr. Prichard differs from the generally-received impression, that monomaniacs are rational except on one particular topic.

"Cases are indeed on record, which, if faithfully related, fully come up to this description. In general, the real character of monomania is very different. The individual affected is, under ordinary circumstances, calm, and exhibits no symptom of that perturbation and constant excitement which are observed in raving madness. But on careful inquiry it will be found that his mind is in many respects in a different condition from that of perfect health. The habits and disposition have, perhaps, been long, in a greater or less degree, in the state which characterizes moral insanity. If we advert to the order and connexion of morbid phenomena, we often learn that on a settled and habitual melancholy, or on a morose and sullen misanthropy, long growing and indulged, or on some other disordered and perverted state of the feelings and affections, a particular illusion has more recently supervened. An individual of melancholic temperament, who has long been under the influence of circumstances calculated to impair his health, and call into play the morbid tendencies of his constitution, sustains some unexpected misfortune, or is subjected to causes of anxiety; he becomes dejected in spirits, desponds, broods over his feelings till all the prospects of life appear to him dark and comfortless. His inclinations are now so altered that no motive has sufficient influence over him to rouse him to voluntary and cheerful exertion. During this period, if questioned as to the causes of his mental dejection, he will probably assign no particular reason for it. At length his gloom and despondency becoming more and more intense, his imagination fixes upon some particular circumstance of a distressing nature, and this becomes afterwards the focus round which the feelings which harass him concentrate themselves. This circumstance is often some real, occasionally some trifling act of delinquency, for which the individual expresses the strongest and perhaps disproportionate self-condemnation. In other instances an unreal phantom suggests itself, in harmony with the prevalent tone of the feelings, which at first haunts the mind as possible, and is at length admitted as reality. Other individuals begin by indulging morose and unfriendly sentiments towards all their acquaintance, magnifying in imagination every trifling neglect into a grievous contumely. They fancy, at length, that they find in some casual occurrence glaring proofs of premeditated designs to ruin them, and expose them to the contempt and derision of society. The disease in these cases has its real commencement long before the period when the particular illusion, which is only an accessory symptom, is discovered, and even before it became impressed on the imagination; but it is not until that impression has taken place that the case assumes the proper character of monomania." 29.

We think it probable that Dr. Prichard is correct in this statement. The monomaniac is *so* rational on all other points but that of his own peculiar illusion, that any little eccentricity or deviation from the normal state of reason is not perceptible, unless the history of the case is correctly known, and the conduct of the individual minutely scrutinized.

Among the varieties of monomania, the worst instances are those in which the thoughts are directed towards the evils of a future life. The unseen state opens the most ample scope for dark and gloomy anticipations, and is selected by the desponding monomaniac as a field for the exercise of his self-torturing imagination. Two centuries ago, persons were every where found who fancied themselves possessed by devils, as the ancients were pursued and agitated by furies. Jacobi informs us that this is still a prominent form of monomania in some Catholic countries. The fear of disease, or hypochondriasis, is only a primary stage of monomania.

In a third section, Dr. P. illustrates moral insanity and monomania by the detail of specific cases. These of course we must pass over. They are collected from various sources, as well as from personal observation, and they are sufficiently illustrative of the species of insanity intended to be delineated.

Section IV. treats of mania, or raging madness. This is distinguished, without difficulty, from the preceding forms of the disease.

"This form of madness generally makes its appearance and reaches its highest pitch more rapidly than others, and it is termed accordingly, when that is the case in a marked degree, *acute mania.* There are, however, in most instances premonitory symptoms, lasting for some days or even for some weeks, before the existence of mania becomes fully established. During this period the individual experiences occasional fits of excitement and confusion, by which his understanding is disturbed. He passes some days in a state of feverish agitation and general uneasiness: he is full of activity, and displays a morbid energy in the pursuits on which he is intent, in which, however, he performs nothing; his projects are for the most part trifling and absurd. He neglects food, loses his appetite, passes sleepless nights, either lying awake and fatiguing his mind with anxious speculations, or rising often, walking to and fro in a state of uneasiness and perturbation. At length his reason is found to be disordered; he appears scarcely to know what he says, talks nonsense, repeats his words frequently, is unable to complete the sentences which he begins, and makes ineffectual efforts to recollect his thoughts, utters rapid and confused expressions in an impetuous manner; cries, laughs, appears irritable and prone to anger, though, perhaps, naturally of mild and sedate temper; is impatient of the most trifling opposition, and absurdly obstinate and capricious; expresses his feelings with an unreasonable degree of warmth and enthusiasm. It is often remarked by their relatives and others, who have an opportunity of observing the conduct of individuals thus affected, that the state of their minds resembles that of persons intoxicated, and that their attempts to collect their thoughts and express themselves with correctness are like the efforts of men half drunk to continue conversation and prove themselves to be sober. The morbid state of a person under these circumstances is always apparent to those who surround him; but it is sometimes doubted whether he is completely mad and a proper subject for restraint, until some attempt being made to oppose him and interfere with his wild pursuits, he breaks out into a degree of violence which obviously requires coercion, and sometimes, though this is not a constant phenomenon in mania, shews that he has laboured under the influence of an unperceived delusion, an insane and absurd impression as to his own person, or his relation to others. Such an impression, if it exists, is not found to be a permanent delusion, like that of the monomaniac; it is soon forgotten, or gives way to some other phantasm.

The disease generally increases in violence, and is several days, or perhaps weeks, before it reaches its highest degree of intensity. During this period the phenomena of derangement vary according to the predominance of particular feelings. In some fear, in others anger, in all some violent emotion has the sway. Many individuals are seized occasionally with painful agitations, with fits of terror; they are under apprehension of some undefined evils impending over them; they pass sleepless nights, and cannot even lie in their beds; and by day they are in a constant state of uneasiness and restless action. Others break out frequently into the most violent expressions of rage and enmity against their relations, who are under the necessity of exercising more or less of restraint and resistance to their absurd proceedings, and have, perhaps, threatened to put them into confinement, or have even carried the proposal into effect. They utter imprecations against those persons who have deprived them of their liberty, declare that they will obtain vengeance, and bring them to condign punishment. The nearest relatives and the most affectionate friends of the lunatic are now among the objects of his most vehement displeasure. As the disorder approaches its highest pitch, the current of ideas becomes more and more turbid; the thoughts and feelings are expressed with cries and ejaculations, with agitation displayed in the manners and countenance, with violent and irregular movements and gestures; the internal sentiments or feelings so absorb the attention that the patient becomes almost unconscious of external impressions. Many individuals abandon in their own persons all regard to cleanliness and decency, and become filthy and disgusting in the extreme. All the functions of the body are in these circumstances of the disease affected; the bowels are irregular, the tongue is furred, the skin cold and clammy. The patient excreates saliva mixed with mucus. His features become haggard and maniacal; his eyes watery and suffused. In some instances the countenance of the individual is so much altered in expression, that his nearest relatives would scarcely recognise him." 74.

Dr. Prichard is not content with this graphic description, but he introduces others from Pinel, Chiaruggi, &c. which only swell the work, without adding to its value.

SECTION 5, treats of the phenomena attendant on the chronic or protracted stage of the disease. The ultimate tendency, he observes, of insanity is to pass into a state of mental decay, or obliteration of the intellectual faculties, afterwards to be described under the head of DEMENTIA. Before this mournful climax of human woe is attained, there is generally an intermediate stage of uncertain duration—which may be termed the chronic period of insanity. This is the state in which most inmates of lunatic asylums are found. It sometimes lasts for many years, or for life.

"The memory, the judgement, the powers of attention and of combination are so much impaired, that the individual is wholly inadequate to the duties of society, and incapable of any continued conversation; his actions and conduct are without steadiness and consistency; his thoughts are deficient in concentration and coherence. In fact this may be considered as a prelude or commencement of that state of incoherence or dementation which will be described in the sequel. It is a combination of the phenomena of mania or monomania with those of dementia." 79.

SECTION 6. *Incoherence or Dementia.*—This is a peculiar and well-marked form of the malady. The mind is occupied, without ceasing, by unconnected thoughts and evanescent emotions—is incapable of continued attention and reflection, and, at length, loses the faculty of distinct perception or apprehension.

"Incoherence is either a primary disease, arising immediately from the agency of exciting causes on a constitution previously healthy, or it is a secondary affection, the result of other disorders of the brain and nervous system, which, by their long duration or severity, give rise to disease in the structure of those organs. The causes which produce the state of incoherence as an original disorder are nearly the same with those which in other cases excite madness in the first instance; they are such agents as break down the powers of the mind by their overwhelming influence, or destroy them by vehement emotions. Secondary incoherence or dementia follows long-protracted mania, attacks of apoplexy, epilepsy or paralysis, or fevers attended with severe delirium. This decay of the faculties has been termed fatuity or imbecility, and it has been confounded with idiotism, which in all its degrees and modifications is a very different state. The distinction, which is very important, has not always been kept in view by writers on disorders of the mind, and even in the works of Pinel we find it sometimes overlooked. M. Esquirol has the merit of having drawn more accurately the line of discrimination. He refers to dementia all the cases of effete or obliterated intellect which are the results of maniacal or other diseases, and are incident to persons originally possessed of sound faculties, and includes those defects only under idiotism, which are original or congenital. 'The imbecile,' he observes, 'have never possessed the faculties of the understanding in a state sufficiently developed for the display of reason. The victim of dementia was once endowed with them, but has lost this possession." 86.

A variety of disorders are occasionally complicated with insanity; but paralysis is so frequently found in connexion with the disease, that Dr. P. dedicates the seventh section of this Chapter to the subject. Often in the advanced stages of insanity a peculiar modification of paralysis presents itself—especially when the cases are passing into the state of dementia. M. Esquirol first drew the attention of the profession to this state, and pointed out its incurability. "It has a peculiar course, says he, ever increasing as the powers of the mind diminish." Whenever this appears, the insanity soon afterwards passes into dementia. It is more frequent in males than in females—in one establishment, the proportions were 95 out of 109—in another, 95 to 14. Three degrees of this paralysis are drawn by M. Calmeil, and copied by Dr. Prichard. We shall introduce the first and third degrees.

"*First Degree.*—An impediment in the movements of the tongue is the first indication of this form of paralysis, and it has often become already very apparent while no embarrassment is as yet discovered in the movements of the limbs. The articulation is no longer perfect, the patient is obliged to use effort in speaking, his words are uttered tardily and with a sort of mumbling and stammering like that of persons intoxicated. If the patient is desired to put out his tongue, he does it without any discoverable deviation; the muscles of the mouth and face preserve their natural position; nothing unusual can be perceived except a kind of muffled articulation, which might escape remark from a person not directing attention to the circumstance." 103.

"*Third Degree.*—Nothing is more deplorable than the aspect of a lunatic affected with general paralysis in the third degree. Those injuries of the brain which affect the intellect and the powers of movement, now approach the very last point. These patients, motionless and insensible, are reduced to a state of mere vegetation; their existence is a kind of slow death. Some individuals are not able to articulate a single word, and only utter vague and confused sounds. The lower extremities are so weak that standing is impossible. A period arrives when even, in sitting, the individual can no longer raise or extend his legs; the hands and arms have not lost so entirely their power of action, but it is evident that they participate in the general weakness. Often there remains no trace of intelligence; food must be put into the patient's mouth; he is totally insensible to excretions, and pays no attention to surrounding objects, and is almost destitute of impressions. He hears indeed, sees, tastes, and perceives strong odours; but is scarcely affected by any sensation.

Digestion, which at first was strong, is at length lost with the other physical powers; the body becomes more and more emaciated; at length the skin sloughs. The lateral parts of the back, the lumbar regions, the cellular tissue which covers the coccyx, the sacrum, the sciatic tuberosities, become the seat of suppuration. Œdema takes place in the depending parts; hectic fever accelerates the exhaustion of physical life." 104.

Section IX. treats of disorders of the physical functions in insanity. He properly observes that other functions besides those of the brain are often deranged—perhaps *always* more or less so, in mania. The secretions, excretions, digestive functions, &c. are frequently disordered. Pinel and others have considered the primary seat of mental alienation to be in the stomach and intestines, from which centre the disease radiated to the organ and functions of the mind. Others, again, have viewed these abdominal disorders as secondary, or consequent on the cerebral affection. There can be little doubt, we think, that the primary cause of insanity commences sometimes in one of these two centres, sometimes in the other. One thing is certain, that they frequently, if they do not generally coexist.

Chap. III.—*On the terminations of insanity,* must conclude our first article of review. The duration of the disease is too various to admit of any calculation—lasting from a few days to many years—so many as to forty or fifty! The proportion of recoveries depends upon various circumstances—attendant on insanity—its simplicity or complication—the character of the alienation—the length of time elapsed—the age, sex, and constitution—and finally, the nature of the causes which have produced the malady. All these items are discussed seriatim, and with considerable minuteness. We can take but a rapid glance at the different sections of this chapter. Where insanity is complicated with disease of the brain, the chances of recovery will evidently be diminished. General paralysis is the worst of all complications. Epilepsy is little less unfavourable to recovery. Mania is more frequently cured than monomania, and the latter is not near so obstinate as dementia. The previous duration is of great consequence in forming the estimate. Dr. Willis affirmed that nine lunatics out of ten recovered, if placed under his care within the first three months. Dr. Burrows, Dr. Finch, Mr. Tuke and others have made nearly similar observations. The most favourable age (according to Esquirol) for recovery, is between the 20th and 30th year. Few are cured after the age of fifty. In respect to sex, many writers consider insanity more curable in women than in men. The summer, too, is more favourable to recovery than other seasons of the year. The term of life in females, is sometimes productive of recovery. It appears that the number of cures effected in the English lunatic asylums is greater than on the Continent. We confess that this does not excite any great wonder in our minds, for many of the continental institutions are a disgrace to the age. There are many exceptions, however, but those who have examined the asylums of Europe will, we apprehend, agree with us in the foregoing remark. It is curious that Bethlem, till very recently, was greatly behind other institutions, in the proportion of cures, notwithstanding the rigid principles of *selection* enforced in that asylum. It has lately improved in this respect.

The RETREAT at York, under Mr. TUKE, exhibits a very favourable feature—and we may add, from personal observation, that there is a small but beautiful RETREAT, near Hackney, under the superintendance of a TUKE, also, where *comfort* is certain, and *recovery* rendered extremely probable.

Dr. P. does not think that insanity "is to be reckoned among the diseases which are very dangerous to life." The state of the brain, though incompatible with sound functions of mind, is yet, he remarks, compatible with healthy functions of the body. We are a little sceptical on this point. Dr. P. himself came to the conclusion that insanity was almost invariably attended by disordered functions, not only in the head, but in the abdominal or glandular organs. These disordered functions, as in cases where there is no insanity, may be compatible with long life—but we question whether the life would not have been still longer if no insanity, and no corporeal function had existed. The number of cases in lunatic establishments, where patients had been lodged there for 30, 40, or even 50 years, cannot satisfy us that insanity curtails life.

"The morbid state of the brain is, however, liable to increase beyond the limit above adverted to, and then the usual phenomena dependent on severe cerebral disease are manifested. It is well known that lunatics are subject in a much greater proportion than other persons to apoplexy, paralysis, convulsions, and all the trains of symptoms depending on different degrees or modifications of cerebral congestion." 147.

This is sufficient to determine a question which is often agitated in life-assurance offices, as to the propriety of insuring insane people, apparently in bodily health, at the usual premiums. We have always set our faces against this proposition, as contrary to sound pathological science. Besides the above consideration, many maniacs die exhausted from the ceaseless excitation of their feelings, and the constant agitation of mind and body which they undergo, added to which is the want of sleep which they so frequently experience. Diseases of the heart, lungs, and abdominal viscera are too often complicated with insanity, to be merely accidental accompaniments.

"In protracted cases death either results from increase in the disease of the brain, which disease up to a certain degree had only interfered with the operations subservient to the mental faculties, but at length becomes incompatible with the merely physical functions of the same organ; or it is the result of accidental disorders, which, owing to the peculiar state of the brain and other organs in lunatics, are more than usually fatal to them." 149.

This brings us to the end of the third chapter, and to a natural division in the series of investigations, where we may pause till next quarter, without any inconvenience to our readers. We could not do justice to Dr. Prichard's labours in one article, unless carried beyond all ordinary bounds; but we hope, in our next number to complete the analysis, to the satisfaction of the author and the professional public in general.

